# Supplementary material for: Identification of cholera hotspots in Zambia: A spatiotemporal analysis of cholera data from 2008 to 2017
Source: PLoS Negl Trop Dis. 2020 Apr 15;14(4):e0008227. doi: 10.1371/journal.pntd.0008227 (PMC7159183; doi:10.1371/journal.pntd.0008227)
Supplement: S1 STROBE Checklist — (DOCX) [file pntd.0008227.s001.docx]

STROBE Statement—checklist of items that should be included in reports of observational studies

|  | Item No | Recommendation |
| --- | --- | --- |
| **Title and abstract** | 1 | (*a*) Indicate the study’s design with a commonly used term in the title or the abstract  It is a spatiotemporal analysis, and included this in the title. |
|  |  | (*b*) Provide in the abstract an informative and balanced summary of what was done and what was found  The abstract contains the sections Methods, Results and Conclusion, where we summarize the results of the paper. |
| Introduction | | |
| Background/rationale | 2 | Explain the scientific background and rationale for the investigation being reported  The first two paragraphs of the Introduction section provide the scientific background and rationale for the investigation. |
| Objectives | 3 | State specific objectives, including any prespecified hypotheses  At the end of the Introduction section, we stated the objective of this study. |
| Methods | | |
| Study design | 4 | Present key elements of study design early in the paper  Key elements of the design are included in the Materials and Methods section. |
| Setting | 5 | Describe the setting, locations, and relevant dates, including periods of recruitment, exposure, follow-up, and data collection  These have been addressed in the different subsections of the Materials and Method section |
| Participants | 6 | (*a*) *Cohort study*—Give the eligibility criteria, and the sources and methods of selection of participants. Describe methods of follow-up  The participants in this study were those who sought healthcare in a hospital in the event of a diarrhea. All people in a district irrespective of thier demographic characteristics were included in this study. However, this is an aggregated data at the district level, thus demographic characteristics were not an issue in this study. Our surveillance period was 10 years, and we have outlined this in the Method section.  *Case-control study*—Give the eligibility criteria, and the sources and methods of case ascertainment and control selection. Give the rationale for the choice of cases and controls  *Cross-sectional study*—Give the eligibility criteria, and the sources and methods of selection of participants |
|  |  | (*b*) *Cohort study*—For matched studies, give matching criteria and number of exposed and unexposed  *Case-control study*—For matched studies, give matching criteria and the number of controls per case. |
| Variables | 7 | Clearly define all outcomes, exposures, predictors, potential confounders, and effect modifiers. Give diagnostic criteria, if applicable  The definition of the outcome has been described in the subsection “Cholera data” and the predictors in the subsection “Water, Sanitation, and Hygiene (WASH)” under the Materials and method section. |
| Data sources/ measurement | 8* | For each variable of interest, give sources of data and details of methods of assessment (measurement). Describe comparability of assessment methods if there is more than one group  We have described the data sources in the 2^nd^, 3^rd^, 4^th^, and in the 5^th^ subsections of the Materials and methods section. |
| Bias | 9 | Describe any efforts to address potential sources of bias  NA |
| Study size | 10 | Explain how the study size was arrived at  This was a retrospective analysis of all available spatiotemporal data on cholera in Zambia. |
| Quantitative variables | 11 | Explain how quantitative variables were handled in the analyses. If applicable, describe which groupings were chosen and why  These have been explained in the subsections “Hotspot identification” and “Statistical analysis of the potential risk factors” under the Materials and Method section. |
| Statistical methods | 12 | (*a*) Describe all statistical methods, including those used to control for confounding  Please see item #11. |
|  |  | (*b*) Describe any methods used to examine subgroups and interactions  NA |
|  |  | (*c*) Explain how missing data were addressed  NA |
|  |  | (*d*) *Cohort study*—If applicable, explain how loss to follow-up was addressed  *Case-control study*—If applicable, explain how matching of cases and controls was addressed  *Cross-sectional study*—If applicable, describe analytical methods taking account of sampling strategy |
|  |  | (*e*) Describe any sensitivity analyses  NA |

Continued on next page

| Results | | |
| --- | --- | --- |
| Participants | 13* | (a) Report numbers of individuals at each stage of study—eg numbers potentially eligible, examined for eligibility, confirmed eligible, included in the study, completing follow-up, and analysed  These have been described in the 1^st^ two paragraphs of the Results section. |
|  |  | (b) Give reasons for non-participation at each stage  NA |
|  |  | (c) Consider use of a flow diagram  NA |
| Descriptive data | 14* | (a) Give characteristics of study participants (eg demographic, clinical, social) and information on exposures and potential confounders  This study used aggregated data, and we have provided information relevant to it in Figure 1. |
|  |  | (b) Indicate number of participants with missing data for each variable of interest  This has been provided in the 1^st^ paragraph of the Results section and in the Table 2. |
|  |  | (c) *Cohort study*—Summarise follow-up time (eg, average and total amount)  This has been provided in the Figures 2 & 3. |
| Outcome data | 15* | *Cohort study*—Report numbers of outcome events or summary measures over time  This has been provided in the Figures 2 & 3. |
|  |  | *Case-control study—*Report numbers in each exposure category, or summary measures of exposure |
|  |  | *Cross-sectional study—*Report numbers of outcome events or summary measures |
| Main results | 16 | (*a*) Give unadjusted estimates and, if applicable, confounder-adjusted estimates and their precision (eg, 95% confidence interval). Make clear which confounders were adjusted for and why they were included  These have been provided in entire Results section and in the Tables 1 to 4. |
|  |  | (*b*) Report category boundaries when continuous variables were categorized |
|  |  | (*c*) If relevant, consider translating estimates of relative risk into absolute risk for a meaningful time period  NA |
| Other analyses | 17 | Report other analyses done—eg analyses of subgroups and interactions, and sensitivity analyses  NA |
| Discussion | | |
| Key results | 18 | Summarise key results with reference to study objectives  This has been stated in the Author Summary |
| Limitations | 19 | Discuss limitations of the study, taking into account sources of potential bias or imprecision. Discuss both direction and magnitude of any potential bias  We have described the limitations and how it affected our study in the 4^th^ paragraph of the Discussion section. |
| Interpretation | 20 | Give a cautious overall interpretation of results considering objectives, limitations, multiplicity of analyses, results from similar studies, and other relevant evidence  We have provided this in the 5^th^ paragraph of the Discussion section. |
| Generalisability | 21 | Discuss the generalisability (external validity) of the study results  We have described this in the final paragraph of the Discussion section |
| Other information | | |
| Funding | 22 | Give the source of funding and the role of the funders for the present study and, if applicable, for the original study on which the present article is based  We have provided these information in the Acknowledgement section and in the Role of funding agencies section. |

*Give information separately for cases and controls in case-control studies and, if applicable, for exposed and unexposed groups in cohort and cross-sectional studies.

**Note:** An Explanation and Elaboration article discusses each checklist item and gives methodological background and published examples of transparent reporting. The STROBE checklist is best used in conjunction with this article (freely available on the Web sites of PLoS Medicine at http://www.plosmedicine.org/, Annals of Internal Medicine at http://www.annals.org/, and Epidemiology at http://www.epidem.com/). Information on the STROBE Initiative is available at www.strobe-statement.org.
