# Supplementary material for: Identification of cholera hotspots in Zambia: A spatiotemporal analysis of cholera data from 2008 to 2017
Source: PLoS Negl Trop Dis. 2020 Apr 15;14(4):e0008227. doi: 10.1371/journal.pntd.0008227 (PMC7159183; doi:10.1371/journal.pntd.0008227)
Supplement: Attachment — Submitted filename: Responses to the reviewers comments.docx [file pntd.0008227.s003.docx]

Responses to the Editor’s and Reviewers’ comments

**Editor’s comments**

Please also address the following re: case definition on lines 105-109. The case definition is unclear. Please describe the inclusion / exclusion criteria for cases, including age-restrictions and whether cases needed to be culture confirmed or just "suspected." Also, it appears that patients under 5 were not included in the study. Please explain and consider adding this as a limitation. The word, "should" on line 108 suggests the case definition may have not been followed. Please comment.

**Response: We have revised the entire section of “Cholera data” (Lines 111-120).**

**In the study limitation section, we have added this “**Secondly, we did not include acute watery diarrhea cases less than 5 years old from areas where cholera was not known to have occurred or acute watery diarrhea cases less than 2 years old from areas where cholera was known to have occurred. Therefore, we could have missed some cholera cases in our study. Cholera can occur in these age groups and the decision to not include these may have led to an under-count **(lines 422-426).**

**Reviewer #1**

1. This is a straightforward study with clearly defined aims and appropriate statistical methodologies employed.

**Response: Thank you.**

2. The cholera data section does not explicitly state the spatial resolution of the confirmed/suspected cases until the ethical section. Would it be possible to combine these sections or have the ethical section come immediately after? The cholera data feels lacking in detail about the spatial resolution of the data otherwise.

**Response: We have now added both spatial and temporal resolution of the cholera data in Method section (line 113).**

3. It would also be helpful if the section on population factors (lines 111-115) described the variables used in more detail rather than requiring the reader to wait for the table in the results section.

**Response: We obtained population factors from two difference sources. The percentage of the population living below the poverty index were obtained from the CSO, which has been described under the “Population and socioeconomic data” section (lines 129-131). The other factors were obtained from Zambia Demographic and Health Survey, which have been described under the “Water, Sanitation, and Hygiene data” section (lines 136-143).**

4. The analysis of results is equally straightforward as the methods, although I have a few questions:

- It is unclear what the numbers on the bars represent in Figure 1, as they do not seem to match the numbers mentioned in the preceding text.

**Response: These are the number of cholera affected districts. We have now stated this in the text (line 250-252) as well as in the title of the figure (Figure 1).**

- The peak in 2009/2010 is striking. Do the authors have access to enough temporally refined data to study why this was the case, in terms of environmental/socio-economic factors? It would be helpful at least for the authors to discuss potential underlying reasons for this peak and then relative decline.

**Response: Unfortunately, we do not have more refined temporal data, thus precluded us to go beyond the year-wise analysis. Regarding the peak in 2009/2010, we observed 89% of the suspected and confirmed cholera cases were reported from the Lusaka district. This could be due to prolonged rainfall and flooding during that time period (**[**https://www.who.int/cholera/countries/ZambiaCountryProfile2011.pdf**](https://www.who.int/cholera/countries/ZambiaCountryProfile2011.pdf)**). We have now revised the result section (lines 251 -254) and discussion section (lines 358 - 364) accordingly.**

- This leads me to also wonder if the hotspots found might differ if the authors split the data into, say, two groups - pre- and post-2010 and then re-ran their analysis on these two different time periods.

**Response: We aimed to identify the hotpots in space and time so that an effective intervention strategy can be planned targeting at those hotspots. Therefore, we employed a spatiotemporal model using long time series data. Comparing the hotspots on or before 2010 and after 2010 was not the aim of the study, and doing so would confuse the policy makers for undertaking an effective intervention, thus avoided. However, we produced yearly cholera map (Figure 2) so that the readers could understand spatial variation of cholera over time in the study area.**

5. Figure 5 is of somewhat low resolution/quality.

**Response: The figures were produced by the Geoda software. Unfortunately, the software does not allow to improve its pixel resolution. However, as suggested, we have tried our best to improve the figure in the revised version.**

6. Conclusions and limitations are clearly presented and supported by the study, so I have no major comments here.

**Response: Thank you.**

7. Some editorial modifications are needed throughout, where words are missing for example.

**Response: We have done the editorial modifications as needed throughout the manuscript.**

**Reviewer #2**

1. The objectives of the study was clearly stated, however the data sources and statistical analysis are not well described.

**Response: We have revised the data sources and the statistical analysis section.**

2. Are the cholera data used in this study monthly or yearly and at district level? Please explicitly state the level(s) in which the data was collected.

**Response: The data are presented by year and by district. We have now explicitly stated it in line 113.**

3. The authors provided a generic description of Zero Inflated Negtative Binomial model but did not explicitly relate the model to the current study. They mentioned that “y” is the independent variable. They should clearly state whether it is the counts of cholera cases in each district at a particular time (yearly?). In formulating the model, subscript should be used to allow the reader follow the methods easily.

**Response: Thanks for this observation. We have revised the statistical analyses section (lines 180-199).**

4. Line 137: Provide reference for SaTScan.

**Response: Provided (line 153)**

5. The annotation in Figure 1 is not clear. What does the number on each bar represents? It does not correspond to the numbers on the y axis.

**Response: We have now provided this information both in the text (line 251-254) and in the Figure title.**

6. The maps in Figure 2 are too small. Change the format to 2 maps per row (5 by 2).

**Response: This has been done as suggested.**

7. Table 2: Why is N=72? Since your study is spatiotemporal, you should have more data points than 72 districts times time. Were the 2008-2017 data sets aggregated for ZINB model?

**Response: These are the socioeconomic data of the 72 districts. We indicated in the Data section that we obtained the socioeconomic data only for a single time point (line 134-135). The temporal data on cholera of the 72 districts were cumulated over the study period for the regression analysis (ZINB model).**

8. On line 261, the authors said that “Distance from waterbodies” was the only significant variable. I will be cautious in reporting this as such, the confidence interval in Table 3 did not support this claim.

**Response: We are sorry to say that we forgot to update the table based on the analysis that included neighborhood incidence rate as a covariate in the model for addressing spatial structure of the data. We have now revised the table (Table 3) and text accordingly (line 302-303).**

9. The limitation should also include the use of a single (year) survey, ZDHS 2014 to extract the WASH data when the study period is 2008-2017.

**Response: We have included this as a limitation (lines 433-434).**

10. The manuscript "Identification of cholera hotspots in Zambia: A spatiotemporal analysis of cholera data from 2008 to 2017” will require full language edits. There are grammatical and orthographic errors here and there.

**Response: The manuscript has now been thoroughly edited.**

11. As per my comments above, I find this to be a straightforward study which is of use for Zambian public health efforts to reduce the burden of cholera. My suggestions have been mainly to explore other ways of dividing the data to see if conclusions remain the same. It would be very interesting to see future work include data from the surrounding countries and look into human movement patterns across and within national boundaries to see how these are affecting the persistence of cholera in the region as a whole.

**Response: This is a welcome comment, but we stated above under the response #4 (reviewer #1) that dividing the data would have created confusion for the readers, in particular when we used a spatiotemporal model to identify the hotspots in space and time. However, we agree with the reviewer that it is important to conduct a regional study in future by undertaking neighboring countries to understand the cross-border problem through movement of the population.**

12. My major concern with this manuscript is the statistical methods used. Rather than using a range of regression models, a multilevel model (ZINB or ZIP with random effects) will suffice. The spatial heterogeneity of the cholera outbreaks will be captured by the random effects. Alternatively, a spatial ZINB or ZIP can be used with a spatially correlated errors. See for example, Loquiha O, Hens N, Chavane L, Temmerman M, Osman N, Faes C, Aerts M. Mapping maternal mortality rate via spatial zero-inflated models for count data: A case study of facility-based maternal deaths from Mozambique. PloS one. 2018 Nov 9;13(11):e0202186.

**Response: We appreciate this comment. However, we note that there are differences between our study and one the reviewer has mentioned where health facilities are nested within the district. The data in our study are not hierarchical, thus a multilevel model is not applicable for it. Since our data are spatially structured, we performed the spatial regression to account for the spatial structure of the data. Also, modelling the data using a Bayesian approach requires setting appropriate prior. Since the prior is often unknown, investigators use a non-informative prior. And, we are not sure whether a Bayesian approach that uses a non-informative prior would be better than the frequentist approach, as we were not aware of any head-to-head comparison between these two approaches. Since our study data were not hierarchical and since we took care of the spatial structure of the data, we believe that we did not employ an incorrect method to identify the risk factors for cholera incidences in our study.**
