# Supplementary material for: Identification of cholera hotspots in Zambia: A spatiotemporal analysis of cholera data from 2008 to 2017
Source: PLoS Negl Trop Dis. 2020 Apr 15;14(4):e0008227. doi: 10.1371/journal.pntd.0008227 (PMC7159183; doi:10.1371/journal.pntd.0008227)
Supplement: Attachment — Submitted filename: Responses to the Reviewers comments_R2.docx [file pntd.0008227.s004.docx]

Responses to the Editor’s and Reviewers’ comments

**Editor’s comments**

Thank you very much for submitting your manuscript "Identification of cholera hotspots in Zambia: A spatiotemporal analysis of cholera data from 2008 to 2017" for consideration at PLOS Neglected Tropical Diseases. As with all papers reviewed by the journal, your manuscript was reviewed by members of the editorial board and by several independent reviewers. The reviewers appreciated the attention to an important topic. Based on the reviews, we are likely to accept this manuscript for publication, providing that you modify the manuscript according to the review recommendations.

**Response: Thank you for accepting our manuscript for publication.**

**Reviewer #1**

1. **Methods:** The objectives of the study are clearly stated and straightforward, and the types statistical analyses are appropriate.

**Response: Thank you.**

2. Cholera data: What effect might the inclusion of suspected cases have on the analysis, particularly if the goal of the study is to identify vaccine targets rather than zones where sanitation improvements are needed? Are there not other water-borne pathogens in the country? It also isn’t clear why cases were included for patients 5 years or older from areas where cholera was not known to have occurred. What is the justification behind this decision?

**Response: Only the initial cases are laboratory confirmed during an outbreak, and subsequent cases are diagnosed based on the WHO clinical definition. We have refined the wording to more clearly explain that Zambian reporting follows WHO criteria (lines 109-113).**

3. Population and socioeconomic data: In the introduction, the authors state that it is particularly peri-urban areas that are at highest risk for cholera transmission in Zambia. Would it be possible to further refine urban/rural classifications as such? It seems it would be important to identify and target not only those districts which are at risk, but specifically the peri-urban zones within these districts.

**Response: This is an important observation, and it is crucial to many intervention efforts, especially given the limited availability of cholera vaccine. However, the data we obtained from the MoH databases only contain administrative classification to the district level. Sub-district, ward or peri-urban level data were not collected. Future studies should include case-studies at sub-district and ward level facilities to collect data at this level and facilitate more targeted efforts. This has been added as a point in the discussion section (lines 394-400).**

4. Figure 1: Does this figure represent confirmed cases alone, or both confirmed and suspected? It would be good to distinguish between the two in the figure.

**Response: Figure 1 represents cholera cases reported in Zambia in the respective years per the WHO definition, which includes both suspected and confirmed cases (only initial cholera cases are confirmed, per WHO, once an outbreak is confirmed, a clinical diagnosis using WHO standard case definition is sufficient to diagnose cholera).**

Furthermore, it seems as though cholera rates have declined overall since the peak in 2010. Can the authors comment on the reasons underlying this pattern? Given this pattern, it would be interesting to see analyses that focus on the data from recent years and compare it to the distribution of previous years. Have the patterns changed, or do they remain the same as for the peak years? If they have changed, what are the implications for vaccine targeting?

**Response: Like many other countries, cholera in Zambia follows a cyclic pattern (see figure below for cholera cases over time). The reason for this cyclic pattern of cholera is not clearly known; however, one explanation could be the infection-derived immunity among the community people which sustains for several years. However, since the hotspots are considered as the source of infection, it is important to undertake the intervention program in these hotspots, so that vaccination is not only control cholera in the community but could also act as barrier for further transmission of the infection to other areas from the source.**

***Curtesy : ZNPHI, 2019***

Number of cases

Case Fatality Rate (%)

| Year | Cholera Cases (#) |
| --- | --- |
| 1978 | 263 |
| 1979 | 12 |
| 1980 | 57 |
| 1981 | 14 |
| 1982 | 1403 |
| 1983 | 233 |
| 1984 | 0 |
| 1985 | 0 |
| 1986 | 0 |
| 1987 | 0 |
| 1988 | 0 |
| 1989 | 44 |
| 1990 | 3717 |
| 1991 | 13154 |
| 1992 | 11659 |
| 1993 | 6766 |
| 1994 | 0 |
| 1995 | 0 |
| 1996 | 2172 |
| 1997 | 36 |
| 1998 | 171 |
| 1999 | 13511 |
| 2000 | 4504 |
| 2001 | 3109 |
| 2002 | 339 |
| 2003 | 1049 |
| 2004 | 12149 |
| 2005 | 1503 |
| 2006 | 5360 |
| 2007 | 2286 |
| 2008 | 2061 |
| 2009 | 4712 |
| 2010 | 6794 |
| 2011 | 330 |
| 2012 | 198 |
| 2013 | 0 |
| 2014 | 0 |
| 2015 | 0 |
| 2016 | 1434 |
| 2017 | 1927 |
| 2018 | 4117 |

|  | Case Fatality Rate (%) |
| --- | --- |
| 1978 | 0 |
| 1979 | 0 |
| 1980 | 0 |
| 1981 | 0 |
| 1982 | 9.34 |
| 1983 | 8.15 |
| 1984 | 0 |
| 1985 | 0 |
| 1986 | 0 |
| 1987 | 0 |
| 1988 | 0 |
| 1989 | 0 |
| 1990 | 8.47 |
| 1991 | 8.29 |
| 1992 | 7.83 |
| 1993 | 6.3 |
| 1994 | 0 |
| 1995 | 0 |
| 1996 | 1.93 |
| 1997 | 0 |
| 1998 | 0 |
| 1999 | 3.96 |
| 2000 | 6.73 |
| 2001 | 5.31 |
| 2002 | 3.54 |
| 2003 | 2.76 |
| 2004 | 3.07 |
| 2005 | 0.47 |
| 2006 | 3.41 |
| 2007 | 1.53 |
| 2008 | 1.89 |
| 2009 | 1.7 |
| 2010 | 0.91 |
| 2011 | 2.12 |
| 2012 | 1.01 |
| 2013 | 0 |
| 2014 | 0 |
| 2015 | 0 |
| 2016 | 0 |
| 2017 | 2.28334 |
| 2018 | 2.76901 |

5. Figure 2: There appears to be a colour in the map that is not represented in the legend (green).

**Response: Thanks for noticing this. We have corrected it.**

6. Results**:** paragraph beginning Line 263: It is interesting to see that some wards were not hotspots within districts, and is pertinent to my comment about identifying peri-urban areas above. Were these wards peri-urban?

**Response: No, not all of those non-hotspots wards are peri-urban. We agree with the reviewer that identifying hotpots at a micro-scale is important, and we highlighted this issue in the text (lines 294-400).**

7. **Conclusions:** Conclusions are well supported by the data, and limitations are addressed albeit briefly. There is clear public health relevance of this study, but maps showing specific wards that should be targeted for vaccines would make this even clearer. Especially if the goal is specifically to focus limited vaccine resources.

**Response: Thanks for the comments.**

8. **Editorial and Data Presentation Modifications:** There are minor edits needed throughout for missing words and clarity.

**Response: We have made necessary edits throughout the manuscript.**

9. **Summary and General Comments:**  This paper is of public health interest for Zambia, and has implications for global cholera control as well. I believe it will be even stronger if analyses are split by time period (distinguishing recent years), and if greater attention is paid to ward-level outputs and peri-urban status.

**Response: Thank for this valuable comment. Regarding split of the time period, we responded previously that we aimed to identify the hotpots in space and time so that an effective intervention strategy can be planned to target at those hotspots. Therefore, we employed a spatiotemporal model using long time series data. Comparing the hotspots on or before 2010 and after 2010 was not the aim of the study and doing so would confuse the policy makers for undertaking an effective intervention, thus avoided. However, we produced yearly cholera map (Figure 2) so that the readers could understand spatial variation of cholera over time in the study area.**

**Regarding the ward-level output, please see our response in #6.**
